# Early Activation of MAP Kinases by Influenza A Virus X-31 in Murine Macrophage Cell Lines

**DOI:** 10.1371/journal.pone.0105385

**Published:** 2014-08-28

**Authors:** Georgetta Cannon, Michelle A. Callahan, Jenny Q. Gronemus, R. Joel Lowy

**Affiliations:** 1 Scientific Research Department, Armed Forces Radiobiology Research Institute, Uniformed Services University of the Health Sciences, Bethesda, Maryland, United States of America; 2 Central Accessing Unit, American Type Culture Collection, Manassas, Virginia, United States of America; Johns Hopkins University - Bloomberg School of Public Health, United States of America

## Abstract

Early molecular responses to Influenza A (FLUA) virus strain A/X-31 H3N2 in macrophages were explored using J774.A1 and RAW 264.7 murine cell lines. NF-kappa B (NFκB) was reported to be central to FLUA host-response in other cell types. Our data showed that FLUA activation of the classical NFκB dependent pathway in these macrophages was minimal. Regulator proteins, IkappaB-alpha and –beta (IκBα, IκBβ), showed limited degradation peaking at 2 h post FLUA exposure and p65 was not observed to translocate from the cytoplasm to the nucleus. Additionally, the non-canonical NFκB pathway was not activated in response to FLUA. The cells did display early increases in TNFα and other inflammatory cytokine and chemokine production. Mitogen activated phosphokinase (MAPK) signaling pathways are also reported to control production of inflammatory cytokines in response to FLUA. The activation of the MAPKs, cJun kinases 1 and 2 (JNK 1/2), extracellular regulated kinases 1 and 2 (ERK 1/2), and p38 were investigated in both cell lines between 0.25 and 3 h post-infection. Each of these kinases showed increased phosphorylation post FLUA exposure. JNK phosphorylation occurred early while p38 phosphorylation appeared later. Phosphorylation of ERK 1/2 occurred earlier in J774.A1 cells compared to RAW 264.7 cells. Inhibition of MAPK activation resulted in decreased production of most FLUA responsive cytokines and chemokines in these cells. The results suggest that in these monocytic cells the MAPK pathways are important in the early response to FLUA.

## Introduction

Despite decades of work on vaccines and antivirals, influenza virus infection remains a major health threat. Influenza and pneumonia were listed as the 8th leading cause of death in the USA for the last several years [1]–[3]. Together, they represent a huge cost to the U.S. economy, estimated in 2005 to be $40 billion [4]. Vaccines are available but require reformulation and re-vaccination on a yearly basis because of virus variability. Optimization of vaccines for new strains can be so time consuming that the influenza season is over before the vaccine can be released [5]. Vaccines are also less effective in influenza susceptible populations such as the very young and the very old. Additionally, antiviral resistance is rising in influenza. There is near complete resistance to M2 channel inhibitors amantadine and rimantadine, and some H1N1 resistance to the hemagglutinin inhibitor oseltamivir [6]–[8]. Host response to infection in target cells is important, and therefore many studies have been conducted on epithelial cells, the primary targets of influenza infection. The response of innate immune cells such as macrophages is also crucial [9], [10] but has been examined in far less detail than the responses of other cell types [11]–[13]. Understanding the specific biochemical pathways controlling influenza induced immune functions such as cytokine and chemokine (CK/CHK) production may lead to the development of improved therapies.

Macrophages present in the intranasal passages are among the first cells to be exposed to an influenza infection. *In vivo* studies show that macrophages are essential to host defense both at early stages of the infection [9] and at later stages to modulate innate and adaptive immune response [14], as well as being important antigen presenting cells [15]. Depletion of alveolar macrophages before infection led to increased morbidity, mortality, and symptom severity in mice indicating how essential macrophage responses are to host defense [16]–[18]. However, they are also responsible for the pathogenesis associated with influenza infections, and there is evidence that macrophage caused tissue damage is a contributing factor to subsequent bacterial infections; a known sequelia leading to morbidity and mortality [19], [20]. The production of a broad array of CK/CHKs characteristic of inflammation is fundamental to macrophage activity both in host defense and pathogenesis [13], [21].

Control of the production of inflammatory CK/CHKs by viral pathogens is broadly attributed to three major cell signaling pathways and the associated transcription factors, NFκB, MAPK and IRF [22]. Influenza virus has been shown to activate all three of these biochemical pathways either directly [23], [24], through inducing production of reactive oxygen intermediates [25], [26], through activating toll like receptors and/or through activating inflammasomes [27], [28]. Activation of both NFκB [13], [27], [29] and MAPK [13], [30] is central to the response, but the role of NFκB is complex. It is suppressed by FLUA NS1 protein [27], [31], and has been shown to be essential for response in many systems [32], but dispensable in others [33]. The majority of studies identifying influenza cellular response elements have been conducted in epithelial cells, MDCK cells, lung alveolar A549 cells, or mouse embryonic fibroblasts, with a smaller number of studies in endothelial and monocytic lineage cells [29], [33]–[35]. The relative importance of the different molecules, particularly the extent of their activation in these other cell types is less clear. Studies with monocytes and some FLUA strains have observed NFκB activation [36]–[38] but a direct linkage to specific CK/CHK production was not reported. A number of studies in human and other species have shown activation of various, but sometimes differing, MAPKs [26], [37]–[41]. Inhibition of p38 in human macrophages [26], [38] or ERK and JNK in swine and avian macrophages [40], [41] was shown to modulate the small subset of the CK/CHKs observed to be induced by influenza in these studies. However, despite the extensive use of murine models to understand influenza pathogenesis, none of the studies that directly link signaling pathways to CK/CHK production in monocytic cells was performed on murine macrophages.

In this study the responses of murine macrophages J774.A1 and RAW 264.7 to a representative “seasonal” virus were assayed by measuring the kinetics of a production of a broad panel of CK/CHKs, canonical and non-canonical NFκB activation, and MAPK activation with an eventual aim of relating the importance of specific signaling molecules’ activation to specific cytokine production in these cells. As CK/CHK responses have been detected as early as 2 h pi [42] we focused on early time points. These macrophage cell lines have been used to study immune response and cell signaling in response to influenza virus [43]–[45] and LPS [46]–[50], and more recently, the ability of high and low pathogenic viruses to reproduce in macrophages [51]. Use of J774.A1 and RAW 264.7 cell lines, both derived from BALB/C mice (from female and male mice respectively) in conjunction with X-31 virus allows us to examine a special state where FLUA strongly drives pro-inflammatory cytokines and chemokines typical of those driven by a variety of strains in a variety of cell types, while minimalizing noise due to individual mice or preparation variances. The lines are classically activated, with J774.A1 cells being in a less differentiated state [52]. The X-31 influenza strain has also been used extensively to study *in vivo* and *in vitro* pathogenesis. We provide evidence that the NFκB canonical pathway was, at best, weakly activated and the non-canonical pathway was not activated at early time points. In contrast MAPK pathways were strongly activated post virus exposure with differing kinetics, levels and duration of activity for JNK1/2, ERK1/2 and p38. The CK/CHKs produced were those (based on a cross comparison of multiple studies *in vitro* in mice and humans and *in vivo* in mice) which are most characteristic of macrophage activation by influenza virus of various strains. The production of the majority of these was strongly inhibited by the MAPK inhibitors. Therefore, MAPK activation appeared to be necessary, and possibly essential, for cytokine/chemokine production by macrophages at early time points post influenza infection in the absence of NFκB activation.

## Materials and Methods

### Viruses and cells

Influenza A virus strain A/X-31 H3N2 was purchased from Charles River Laboratories, Frederick, MD, titered by plaque assay on MDCK cells as previously described [43] except that PBS with 0.9 mM calcium and 0.5 mM magnesium (Invitrogen) with 1% glucose added (Sigma) was used for *in vitro* dilutions and infections. Virus was used at a multiplicity of infection of 1. This moderate MOI was chosen to give a more uniform response over the population of cells. J774.A1 cells and RAW 264.7 cells were obtained from ATCC, Manassas, VA. J774.A1 cells were passaged in RPMI 1640 medium (Invitrogen, Carlsbad, CA) supplemented with 5% heat inactivated fetal bovine serum (FBS, defined, U.S. origin, Hyclone SH30070.03, Thermo Scientific, Pittsburg, PA) and antibiotics (100 U/ml penicillin and 100 µg/ml streptomycin, Invitrogen) in suspension culture. Cells were plated in 6 well plates 18 to 24 h before infection. RAW 264.7 and MDCK cells were cultured in Dulbecco’s modified Eagles medium with 4.5 mg/ml glucose (Invitrogen) supplemented with 10% FBS and antibiotics with l-glutamine (100 U/ml penicillin, 100 µg/ml streptomycin, 2 mM glutamine, Invitrogen). All cells were cultured at 37°C in 5% CO_2_ in a humid atmosphere. Cells were used up to passage 30 and were monitored for consistency of morphology and growth rate. Infections were carried out in 200 µl of phosphate-buffered saline with calcium and magnesium (PBS, Invitrogen) +1 mg/ml glucose (Sigma, St. Louis, MO) +0.2/µg/ml acetylated trypsin (Sigma) for 2 h with periodic hand rocking. Control cells were mock infected. Lipopolysaccharides (LPS) treated cells were mock infected and received 1 µg/ml LPS from *E. coli* serotype 055:B5 (Sigma, St. Louis, MO).

### Cytokine/Chemokine assays

Supernatants were collected from infected and control cells and subjected to single or multi-plex fluid phase antibody based analysis for CK/CHKs using a 22-plex, 12-plex, or single-plex mouse xMAP immunoassay kit following the manufacturer’s instructions (Millipore, St. Charles, MO). Samples were run and analyzed on a Luminex 200 system (Austin, TX) with xPonent software Ver. 3.1. Differences were tested using multiple Student’s two-tailed t-tests assuming equal variances.

### Western blotting

Cells were lysed in ice cold lysis buffer [100 mM Tris-HC1 pH 8.0 at 25°C (Quality Biological Inc., Gaithersburg, MD); 100 mM NaCl (Sigma); 2 mM EDTA (Sigma); 1% (v/v) 10%Igepal CA630 (Sigma); 1 mM sodium vanadate (New England Biolabs, Ipswich, MA); 50 mM sodium fluoride (Sigma); 1X cOmplete mini EDTA free protease inhibitor solution (Roche, Branford, CT)], cleared, quantitated and equal amounts of protein per lane were subjected to electrophoresis followed by blotting to nitrocellulose membranes (Amersham Hybond-ECL, GE Healthcare, Piscataway, NJ). Membranes were probed with the following unconjugated antibodies: anti-IκBα, anti-IκBβ, anti-p100/52, anti-RelB (all from Santa Cruz Biotechnology, Santa Cruz, CA); anti-JNK1/2 pTpY^183/184^, anti-JNK1, anti-ERK1/2 pT^185^pY^187^, anti-ERK1/2, anti-p38 pTpY^180/182^, p38 (all from Invitrogen); anti-H3N2 influenza (Virostat, Portland, ME) and anti-β-actin (Sigma). Peroxidase conjugated secondary antibodies used were AffiniPure F(ab’)_2_ donkey anti mouse for β-actin and AffiniPure goat anti-rabbit IgG, for the remaining antibodies (both from Jackson ImmunoResearch Laboratories Inc., West Grove, PA). Blots were developed with Amersham ECL Advance Western Blotting Detection Kits (GE Healthcare) following the manufacturer’s protocol and imaged with a Fuji LAS 3000 system using Image Reader Software Ver. 2.2. Densitometry was performed with Multigage Ver. 3.1 software. Background was subtracted and loading normalized based on the β-actin control. Densitometry graphs are shown as percent of control with error bars representing ± standard error of the mean.

### Immunofluorescence

Cells were plated on glass chamber slides 24 h before infection and treated as indicated. Slides were washed, fixed in 4% formalin, quenched with ammonia chloride, and the cells were permeabilized with 0.2% Triton X (Sigma). Slides were washed, blocked and stained using a polyclonal rabbit antibody to mouse NFκB p65 (Santa-Cruz) and a Rhodamine Red-X (RRX) conjugated goat anti-rabbit secondary antibody (Jackson ImmunoResearch Laboratories). Slides were examined with a Nikon 300 Eclipse microscope and imaged with a Princeton Instruments CCD-768-K/2 camera and Metamorph Ver.3.5 software (Molecular Devices, Sunnyvale, CA).

### Inhibitors

Cells were treated with inhibitors for 1 h in complete growth media at 37°C prior to all other manipulations and the inhibitors were then maintained in the culture media during and post-infection. Inhibitors used were: JNK inhibitor–SP600125 (EMD Biosciences, Gibbstown, NJ) used at a final concentration of 10 µM [53], [54]; and ERK inhibitor II– FR180204 (EMD Biosciences) used at a final concentration of 30 µM [55], [56].The inhibitors were suspended in DMSO and control cells received a volume of DMSO alone equal to the greatest amount received by the inhibited cells in a given experiment.

### Statistics

Data in all figures and supplemental data tables are from 3–5 independent experiments as indicated by the number (n) and reported as the mean ± the standard error of the mean (SEM). Student’s two-tailed t-tests assuming equal variances were used to determine the significance of changes for MAPK inhibitor effects. A two tailed test was used as *a priori* it was unknown whether there would be an increase or decrease of a particular CK/CHK’s level at a specific time. P-values are reported in the supplemental tables.

## Results

### Canonical NFκB showed limited activation at early time-points in X-31 exposed J774.A1 and RAW 264.7 cells

NFκB has long been implicated in driving pro-inflammatory chemokine and cytokine production in innate immune responses [57]. NFκB transcription factors act as dimers, which are located in the cytoplasm when inactive. In the case of the canonical pathway, activation involves phosphorylation and degradation of the IκB inhibitor subunits. For the non-canonical pathway, the end of one of the dimer partners is phosphorylated and partially degraded. In either case, after the degradation step the transcription factor dimer is released to re-localize to the nucleus and drive gene transcription [58]. In epithelial cells canonical NFκB activation was observed in response to FLUA infection, as shown by the degradation of NFκB inhibitors IκBα and IκBβ visualized by Western blot, by NFκB nuclear translocation visualized by immunofluorescence, or by enhanced transcription factor DNA binding shown by electromobility shift assay or reporter gene assay [34], [59]–[61].

**Figure 1:**
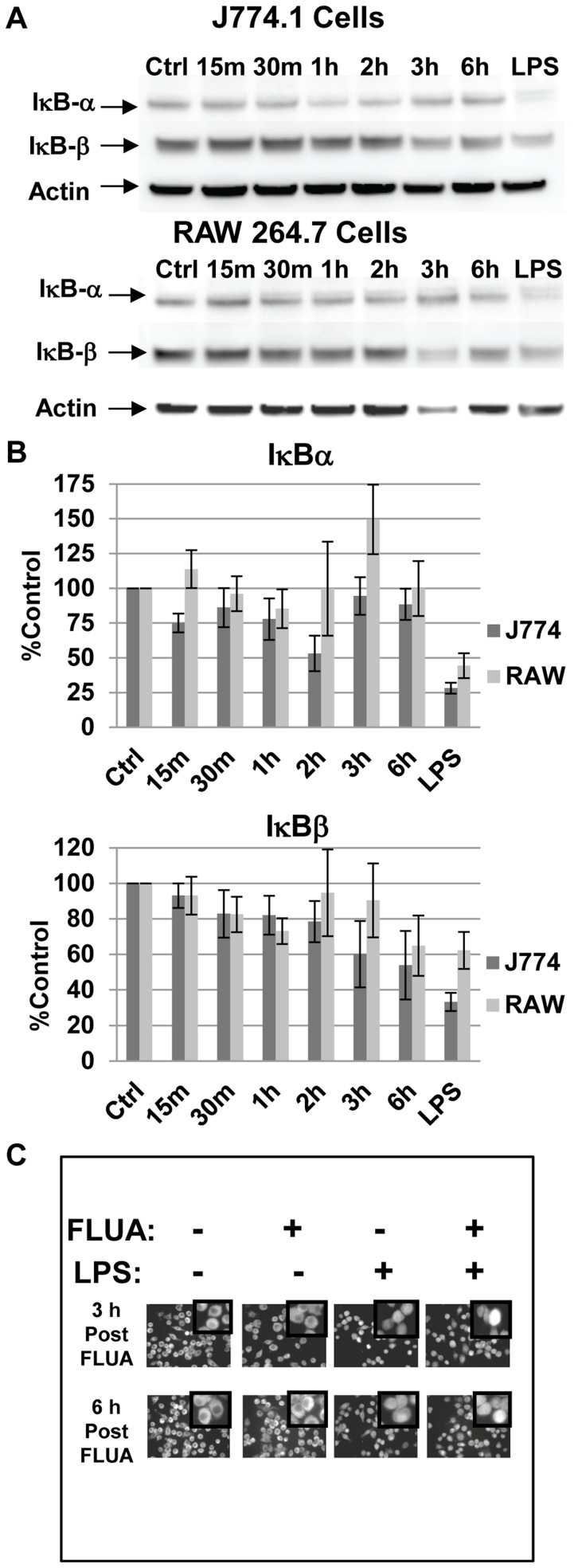
NFkB is not activated by FLUA at early time-points. **A.** In J774.A1 cells IκBα levels were reduced at 2 h while IκBβ levels showed a gradual decline across the time points (but approximated control levels at 2 h). In RAW 264.7 cells, IκBα stayed near or above control levels while IκBβ levels showed a mild decline. Representative Western blots of cell lysates collected at the indicated time points post exposure to X-31 FLUA. Control and LPS treated (1 µg/ml for 15 minutes) cells were mock infected. **B.** Densitometry graphs from the Western blots. Data are normalized to actin, background is subtracted and protein levels are expressed as percentage of the mock infected control. Means are shown ± SEM, n = 3 to 6 independent experiments depending on time point. **C.** Nuclear localization of NFκB subunit p65 is not increased by X-31 infection of J774.A1 cells. Cells were infected or mock infected for the indicated periods, fixed, permeabilized and stained with anti-p65 antibody. LPS cells received 1 µg/ml LPS 30 minutes before fixation. LPS did increase NFκB nuclear localization as indicated by immunofluorescence. LPS activation was not blocked by prior X-31 exposure. 60X scale fields are shown with 255X digitally zoomed insets.

We infected our macrophage cell lines with X-31 and assayed NFκB activation by monitoring levels of IκBα and IκBβ by Western blots. Degradation of IκB subtypes post virus exposure varied slightly between the cell types (Figure 1A). In J774.A1 cells, levels of IκBα decreased transiently at 2 h to 54% of control levels but were above 75% of the control levels for most time points. In RAW 264.7 cells, IκBα degradation was not apparent in response to FLUA. In both cell types IκBβ shows a progressive degradation to about 50–60% of the control levels by 6 h post influenza exposure with a later, milder response seen in the RAW 264.7 cells (Figure 1B). A 15 minute LPS treatment was used as a positive control for IκB subunit degradation as LPS is known to rapidly activate NFκB in these cells. LPS treatment prompted a rapid decrease of IκBα levels to 28% controls levels in J774.A1 cells and 35% for RAW 264.7 cells and 33% and 62%, respectively for IκBβ. We also examined the functional capability of the NFκB sub-unit p65 to localize to the nucleus post influenza exposure, again using LPS treatment as a positive control (Figure 1C). We saw no virus driven p65 activation by immunofluorescence, although LPS did increase NFκB nuclear localization. Additionally, LPS activation was not blocked by prior X-31 exposure. These results showed that the canonical NFκB pathway was not rapidly or robustly activated in response to influenza virus. The expected strong LPS response was observed demonstrating that the limited FLUA induced response was not due to the lack of a functional pathway, or a capacitatively diminished one in these cells. Furthermore the LPS response post FLUA infection demonstrated that the NFκB pathway was not blocked to all signals in infected cells.

### Non-Canonical NFκB was not activated in X-31 exposed J774.A1 and RAW 264.7 cells at early time points

**Figure 2:**
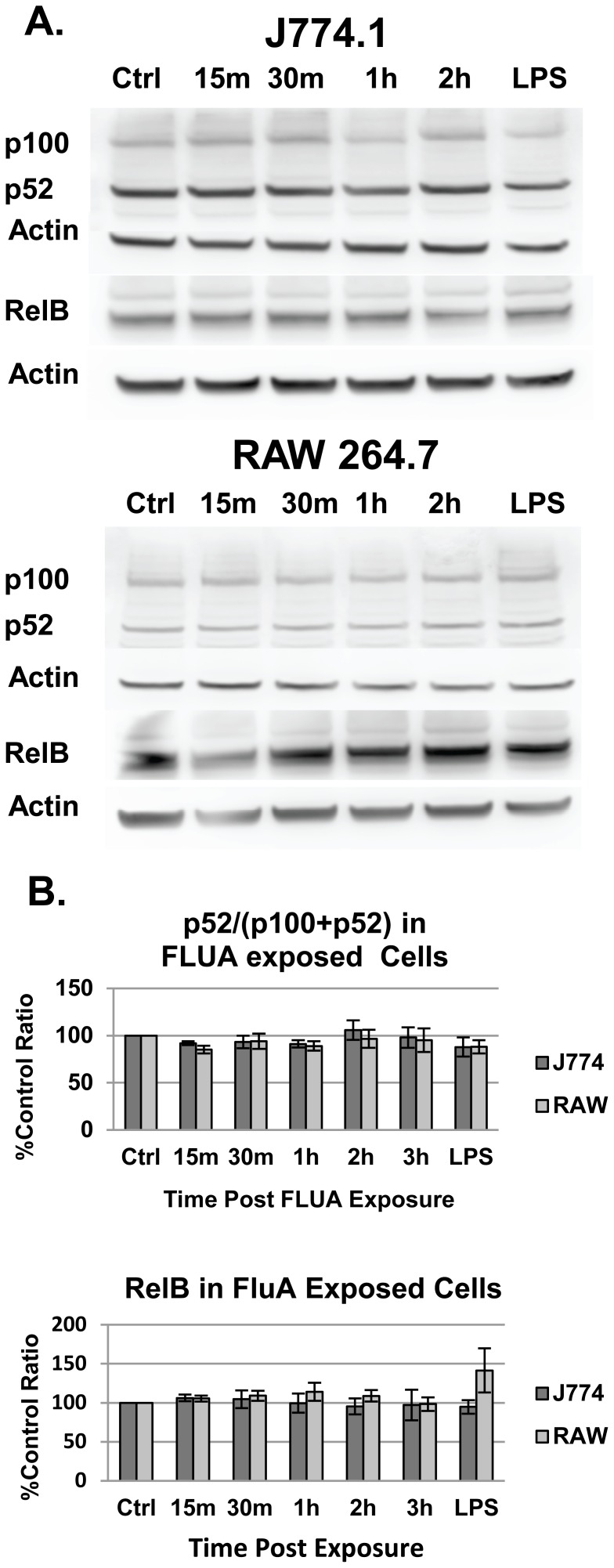
The non-canonical NFκB pathway is not activated by X-31 exposure. The percent of the total p52 and p100 protein in the activated p52 form did not change from that in the control mock infected cells. The expression of RelB, the partner of p52, was also unchanged. **A.** Representative Western blots. **B.** Densitometry graphs from the Western blots. Means are shown ± SEM, n = 4 to 7 independent experiments depending on time point.

Several viruses, including FLUA, have been shown to activate the non-canonical NFκB pathway, albeit usually in conjunction with the canonical one [62]. The non-canonical pathway is typically activated in a slow progression peaking several hours after a differentiation or developmental stimuli. Although we primarily focused on early signaling responses, we also examined this pathway in the event that non-canonical signaling was being used as means of activating NFκB. The levels of both intact p100 and the activated cleavage product p52 were measured post FLUA exposure. The shortened form of p100/p52 did not increase as a percentage of the total protein and it did not vary from the mock infected cells indicating that the pathway was not activated at the time-points examined (Figure 2).

### X-31 infection induced proinflammatory chemokine and cytokine production from J774.A1 and RAW 264.7 cells

**Figure 3:**
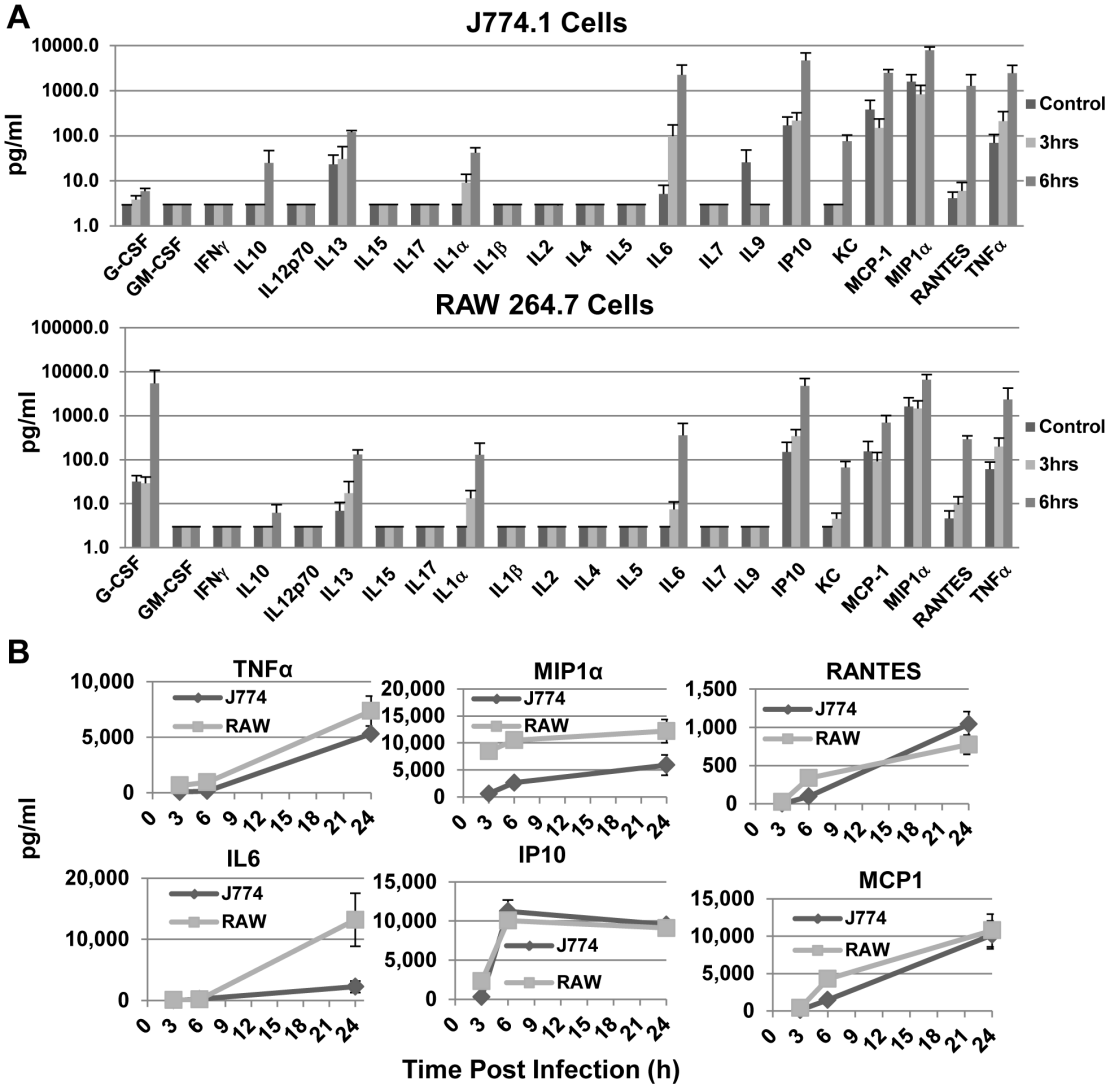
Proinflammatory cytokines are induced by X-31 exposure at early time points post exposure. **A.** Supernatants from cells 3 h or 6 h after exposure to X-31, along with those from mock infected controls, were assayed for CK/CHK by multiplexed fluid phased antibody based analysis. Cytokine concentrations were calculated based on known concentration standards run at the same time. Data shown are the mean ± SEM, n = 5 independent experiments. **B.** Production kinetics of selected CK/CHK induced by FLUA. The charts illustrate commonalities and differences. TNFα and IL6 showed a lag of 3–6 h whereas MIP1α and IP10 were maximal by 3 h. The RANTES and MCP1 responses were intermediate. RAW 264.7 cells responded faster and to higher levels than J774.A1 cells especially at 3 and 6 h pi. Means ± SEM, n = 3 independent experiments.

Macrophages are known to produce CK/CHKs in response to FLUA, most of which are thought to depend on NFκB activation. We previously showed that J774.A1 cells produce TNFα in response to FLUA at 24 h [43]. As these cells did not strongly activate NFκB at early time points, we examined both cells lines for the production of a panel of CK/CHKs 3, 6 and 24 h post X-31 exposure (Figure 3). Since protein production post transcription factor activation typically requires 3–4 h the two earlier time-points should reflect early signaling responses (Figure 3A). These cells produced a wide selection of viral response pro-inflammatory CK/CHKs. Interestingly, chemokines predominated at early time-points, and as would be expected, TNFα and IL6 release also occurred quickly. The kinetics of CK/CHK production for J774.A1 and RAW 264.7 have several different response patterns (Figure 3B) but each individual CK/CHK’s response was similar between the two cell types. TNFα and IL6 show a delayed response, with low levels at 3 and 6 h pi compared to those produced by 24 h pi. MIP1α and IP10 show rapid kinetics with the 6 h level being at or near that seen at 24 h pi. RANTES and MCP-1 illustrate an intermediate response with 6 h amounts being considerably greater than 3 h, but less than the amounts seen by 24 h pi. The major difference between the cell types is a kinetic one, with J774.A1s producing considerably lower quantities at 3 and 6 h for most CK/CHKs. For some but not all, the 24 h concentrations are similar. IL6 is a notable exception as the J774.A1 cells produce much less than RAW 264.7 cells.

The CK/CHKs induced correlated well with those induced by FLUA *in vivo* and *in vitro* in human and mouse primary culture macrophages (Figure S1). Therefore these cells produce a typical panel of inflammatory CK/CHKs at early time points post FLUA infection when NFκB activation is minimal. Next we considered MAPK involvement in the early control of CK/CHK production.

### MAPKs JNK and ERK were rapidly activated by X-31 infection of J774.A1 and RAW 264.7 cells

**Figure 4:**
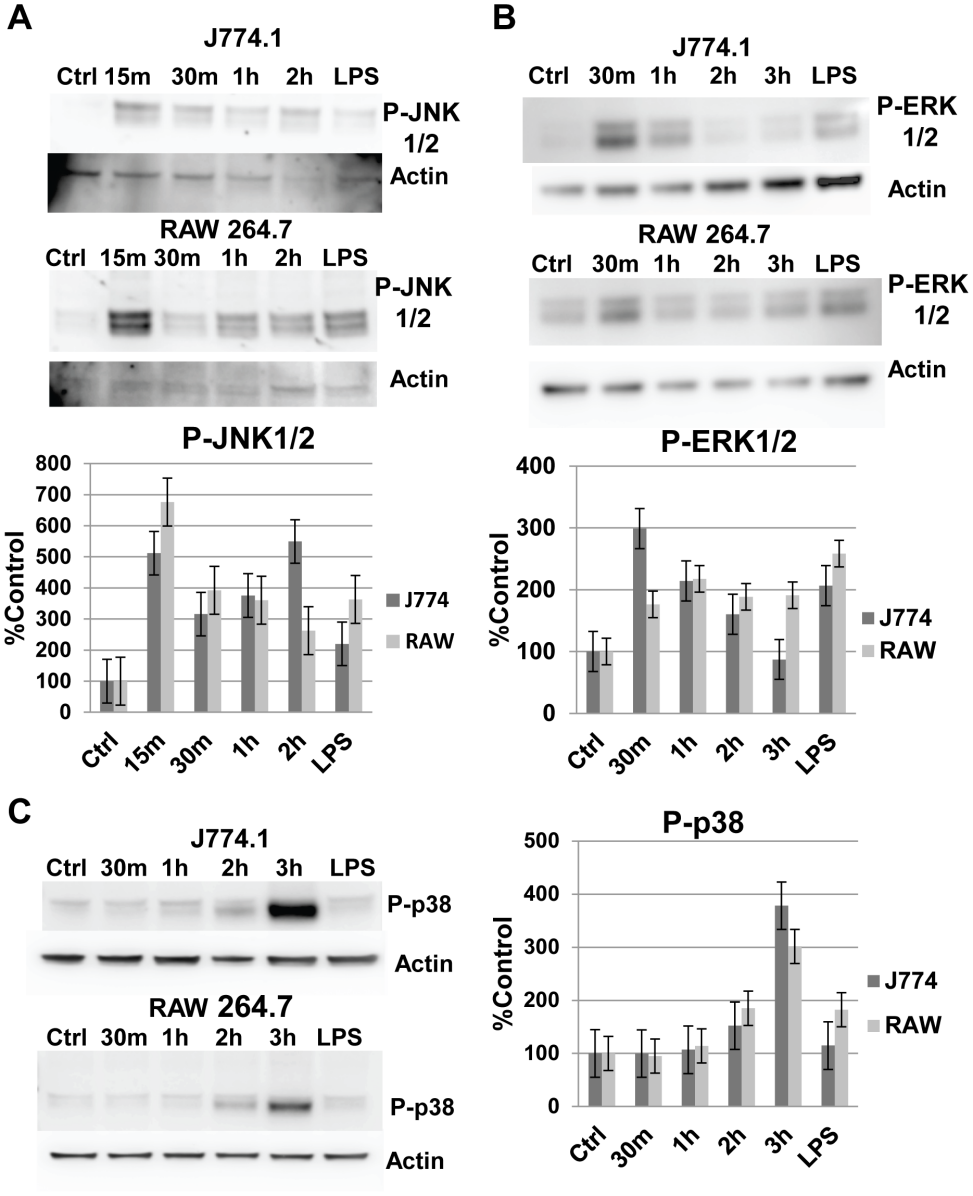
MAPKs are rapidly phosphorylated following FLUA infection. **A.** J774.A1 and RAW 264.7 cells rapidly phosphorylate JNK 1/2 in response to X-31 exposure. In both cell lines maximal response was reached by 15 minutes post virus exposure. Control and LPS treated (1 µg/ml for 15 minutes) cells were mock infected. Representative Western blots and densitometry graphs from the Western blots (data are mean ± SEM, n = 5 independent experiments). **B.** J774.A1 and RAW 264.7 cells rapidly phosphorylate ERK 1/2 in response to X-31 exposure. In J774.A1 cells maximal response was reached by 30 minutes. RAW 264.7 cells had a lower maximal expression that was reached by 1 h. Control and LPS treated (1 µg/ml for 15 minutes) cells were mock infected. Representative Western blots and densitometry graphs from the Western blots (data are mean ± SEM, n = 4 independent experiments). **C.** J774.A1 and RAW 264.7 cells phosphorylate p38 in response to X-31 exposure. In both cell lines maximal response was reached by 3 h, a noticeable delay compared to the JNK1/2 and ERK1/2 activations. The antibody used recognizes α, β and γ p38 isoforms. Control and LPS treated (1 µg/ml for 15 minutes) cells were mock infected. Representative western blots and densitometry graphs from the western blots (data are mean ± SEM, n = 5 independent experiments).

Extensive work in epithelial cells and some other cell types has demonstrated the activation of MAPKs by various FLUA isolates [63]–[66]. The JNK kinase is associated with a wide network of cellular responses ranging from apoptosis to proliferation [67]. In macrophages, JNK has long been implicated in inflammatory gene activation in response to pathogens [68]. JNK activation is also required for macrophage differentiation and proliferation in response to CSF-1 [69]. We examined our cells for JNK phosphorylation/activation in response to X-31 exposure (Figure 4A). In contrast to the NFκB results, JNK was vigorously and rapidly activated, with phosphorylation peaking at 6 to 7 times control levels 15 to 30 minutes post viral exposure in both cell lines. Another MAPK, ERK is implicated in a wide variety of cellular responses including the production of inflammatory cytokines [70]–[72]. In our cell lines, ERK was also rapidly phosphorylated/activated by X-31 exposure (Figure 4B). The magnitude of the response, 2 to 3 fold above control levels, was less than the JNK response particularly in the RAW 264.7 cells, but remained above control levels for 2 (J774.A1 cells) or 3 (RAW 264.7 cells) h. As expected, the positive control response to LPS was also observed.

### MAPK p38 was also activated by X-31 infection of J774.A1 and RAW 264.7 cells, although with slower kinetics

p38 represents the third major type of MAPKs and like the other two is activated in response to a variety of stimuli such as inflammatory cytokines and environmental stresses [73], [74]. It is hyper induced by highly pathogenic influenza A H5N1 virus and its inhibition reduces the expression of TNFα from human monocyte derived macrophages exposed to H5N1 influenza [38]. In our experimental macrophages, p38 was activated by exposure to X-31 although with slower kinetics than those we had seen with JNK and ERK (Figure 4C). By 3 h post exposure the amount of phosphorylated p38 was 3 to 4 times the amount seen in mock infected controls in both cell types.

### Early CK/CHKs responses to X-31 in macrophages is primarily dependent on MAPK activation in the absence of NFκB activation

**Figure 5:**
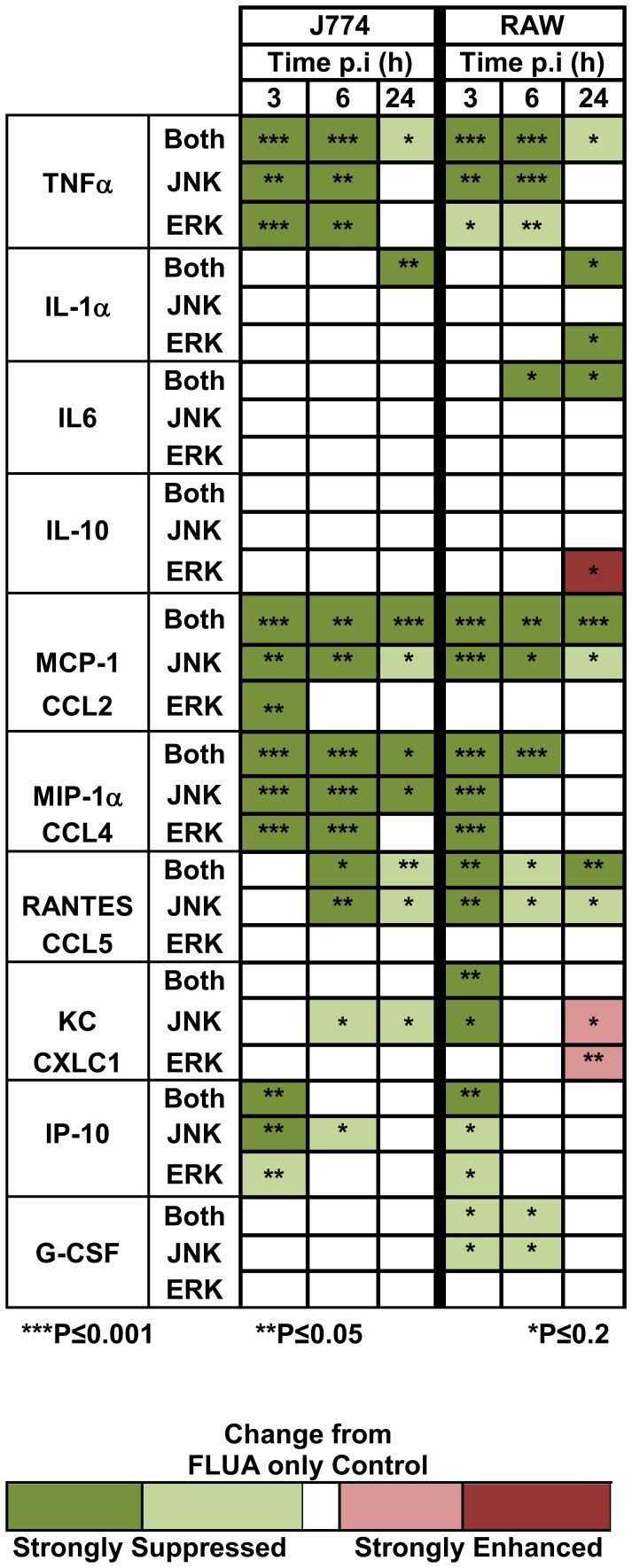
Heat map summary of the effect of MAPK inhibitors on FLUA-induced CK/CHKs. Supernatants from cells pre-treated with the indicated inhibitors for 1 h then exposed to FLUA were collected 3, 6, or 24 h after exposure, along with those from mock infected controls. They were assayed for selected CK/CHK by multiplexed fluid phased antibody based analysis. Cytokine concentrations were calculated based on known concentration standards run at the same time. CK/CHKs are arranged by family, TNF, interleukins, CCL, CXCL, and growth factors and then in numerical order. Colors are based on the amounts produced compared to controls that received FLUA only. Data are means ± SEM, n = 3 independent experiments. P values are indicated by asterisks: ***P≤0.001; **P≤0.05; *P≤0.2 as determined by t-tests. No color indicates there was no significant effect of the inhibitor. Absolute production levels are shown in Figures 6–9, Tables S1 and 2. FO–FLUA only; JEI–both JNK and ERK Inhibition; JI–JNK inhibition; EI–ERK inhibition.

**Figure 6:**
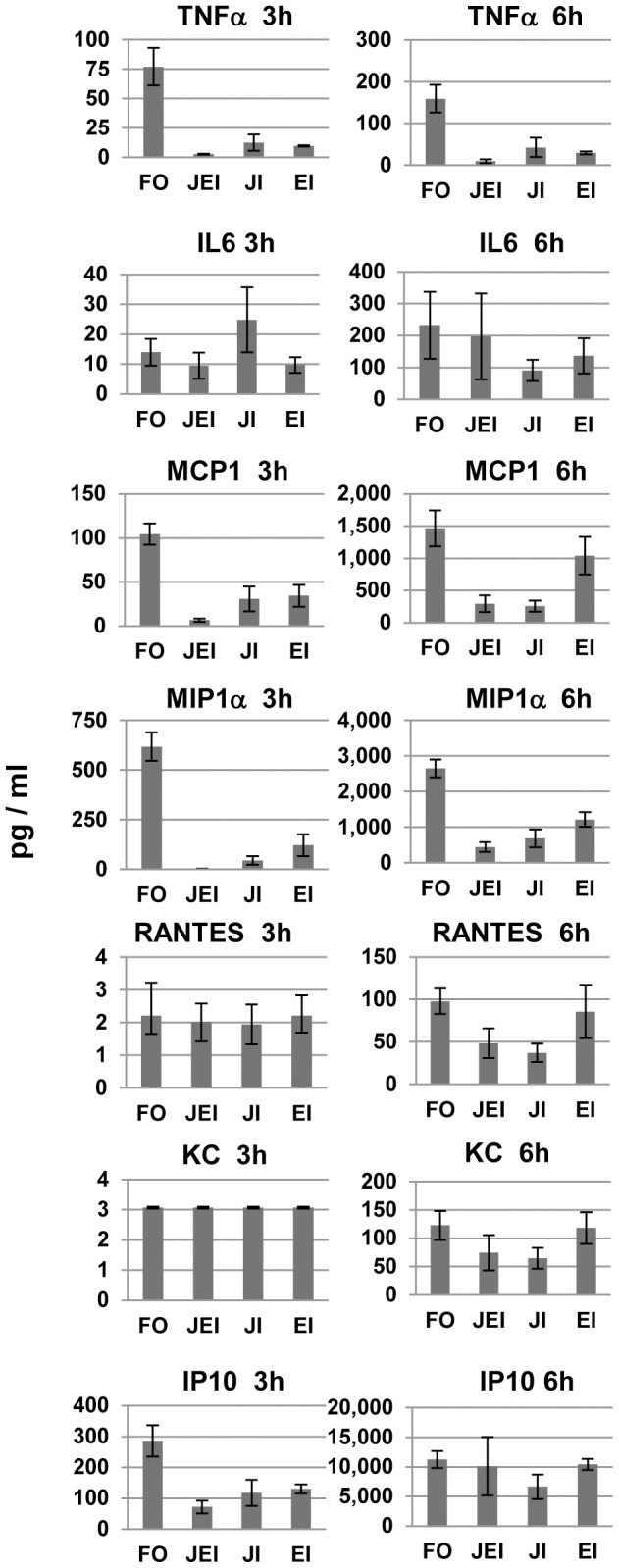
Effects of MAPK inhibitors on FLUA-induced CK/CHKs from J774.A1 cells at short time points. Supernatants from cells pre-treated with the indicated inhibitors for 1 h then exposed to FLUA were collected 3 or 6 h after exposure, along with those from mock infected controls. They were assayed for selected CK/CHK by multiplexed fluid phased antibody based analysis. Cytokine concentrations were calculated based on known concentration standards run at the same time. Data shown are the mean ± SEM, n = 3 independent experiments. FO–FLUA only; JEI–both JNK and ERK inhibition; JI–JNK inhibition; EI–ERK inhibition.

**Figure 7:**
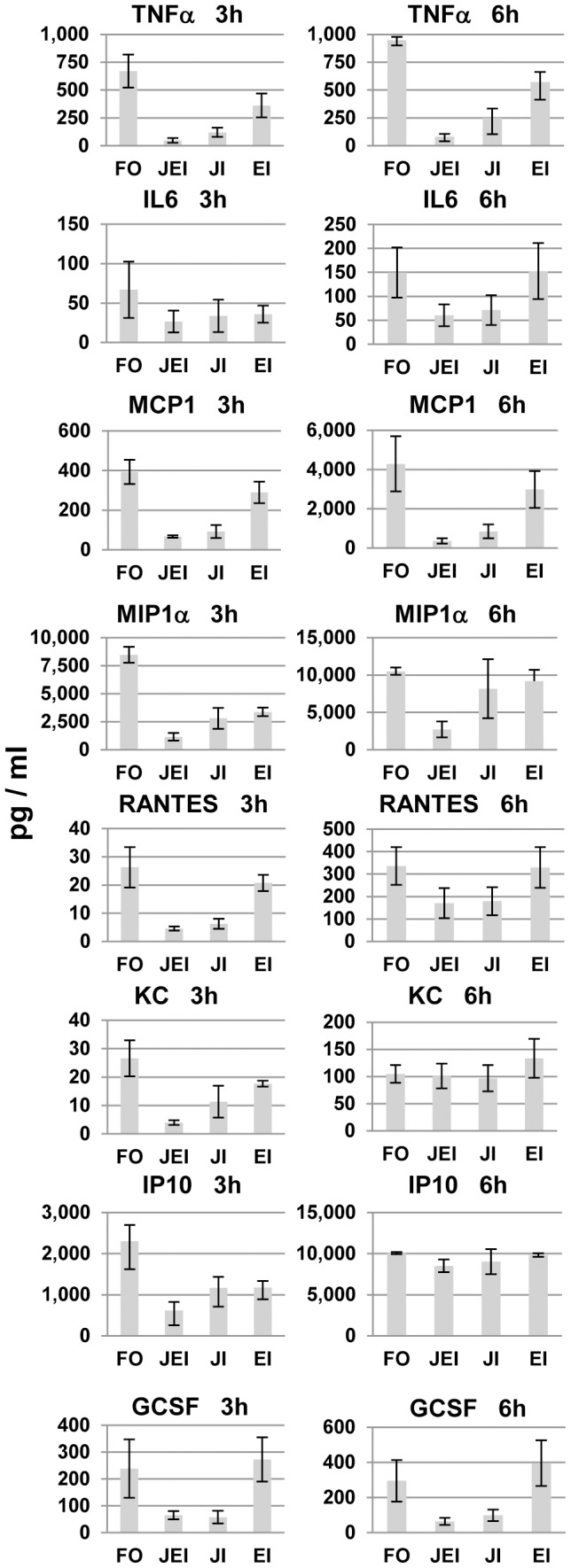
Effects of MAPK inhibitors on FLUA-induced CK/CHK from RAW 264.7 cells at short time points. Supernatants from cells treated with the indicated inhibitors as described in Methods then exposed to FLUA were collected 3 or 6 h after exposure, along with those from mock infected controls. They were assayed for selected CK/CHK by multiplexed fluid phased antibody based analysis. Cytokine concentrations were calculated based on known concentration standards run at the same time. Data shown are the mean ± SEM, n = 3 independent experiments. FO–FLUA only; JEI–both JNK and ERK Inhibition; JI–JNK inhibition; EI–ERK inhibition.

**Figure 8:**
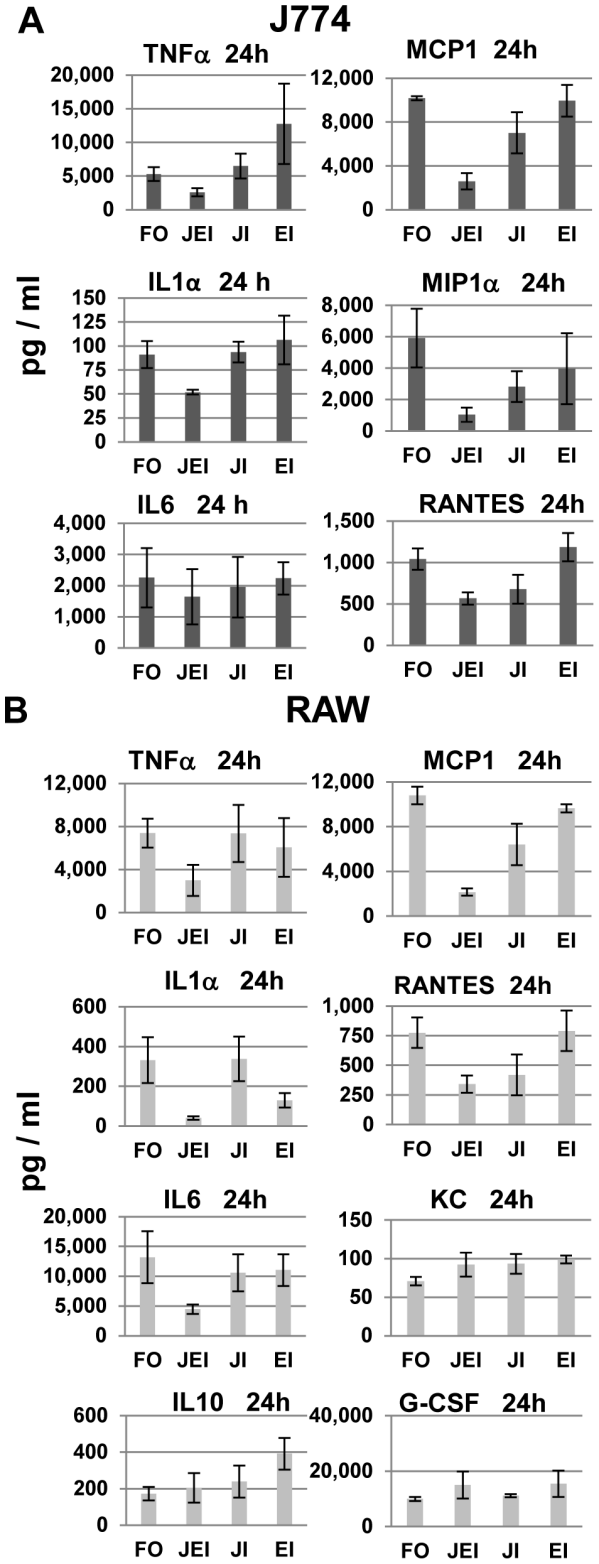
Effects of MAPK inhibitors on FLUA-induced CK/CHK from cell lines at 24 h. Supernatants from cells pre-treated with the indicated inhibitors as described in Methods then exposed to FLUA were collected 24 h after exposure, along with those from mock infected controls. They were assayed for selected CK/CHK by multiplexed fluid phased antibody based analysis. Cytokine concentrations were calculated based on known concentration standards run at the same time. Data shown are the mean ± SEM, n = 3 independent experiments. FO–FLUA only; JEI–both JNK and ERK Inhibition; JI–JNK inhibition; EI–ERK inhibition.

To test the theory that the MAPKs were driving the NFκB independent chemokine/cytokine production we saw after exposure of these cells to X-31, we treated the cells with inhibitors to either JNK1/2 or ERK1/2 or to both. We pretreated with the inhibitors for 1 h, infected the cells and then assayed the effect on FLUA induced CK/CHK production at 3, 6 and 24 h post infection in the continuous presence of the inhibitors (Figures 5–8; Tables S1–S2). The 3 and 6 h time points were chosen to isolate the effects of signaling molecules activated at early time points as opposed to later acting molecules. The 24 h time point tested the durability of the effects.

At 3 and/or 6 h post exposure most of the cytokines induced by FLUA were substantially suppressed by the inhibitors in both cell types as summarized in Figure 5. The most frequent response seen in both cell types was a large reduction in CK/CHK production by JNK inhibitor treatment, with a lesser effect by ERK inhibitor treatment. Generally the greatest suppression occurred when both inhibitors were used in combination. Importantly both untreated and treated cells continued to produce CK/CHKs as absolute concentrations continued to increase regardless of treatment. Therefore the effect of the inhibitors was to decrease the rate of production not to cause a complete block.

In J774.A1 cells, the FLUA induced expression of TNFα, MCP1 and MIP1α was strongly suppressed by the individual JNK and ERK inhibitors and further suppressed by the combination at 3 and 6 h pi (Figure 6 and Table S1). At 3 h, IP10 was also suppressed by each inhibitor and decreased further by the combination, but by 6 h only JNK alone produced mild suppression. RANTES and KC were not detected at 3 h; levels shown are near the limit of the assay. At 6 h there was a modest increase in both which was suppressed by the JNK inhibitor alone. At 24 h pi TNFα, MCP1, MIP1α, and RANTES expression was still suppressed by the combined inhibitors and by JNK inhibition but the proportional inhibition was reduced (Figure 8A, Table S1). MCP1, MIP1α and RANTES levels in ERK inhibitor treated cells were similar to those from untreated cells at 24 h pi. Surprisingly, TNFα levels were increased at 24 h in the presence of ERK inhibition although the increase did not reach significance (Figure 8A). For IL6, IP10, and KC (Figure 8A, Table S1) production levels were equivalent to untreated cells by 24 h pi. J774.A1 cells, like RAW 264.7 cells, did not produce detectable IL1α until 24 h post infection. Only combined JNK/ERK inhibition partially suppressed IL1α expression. J774.A1 cells, unlike RAW 264.7 cells, did not express G-CSF or IL10 in response to FLUA.

In RAW 264.7 cells the suppression of TNFα, MCP1, RANTES, and G-CSF production at both 3 and 6 h post infection was primarily due to the JNK inhibitor (Figure 7, Table S2). KC was inhibited by JNK inhibitor alone at 3 h post exposure but the effect was lost by 6 h. For MIP1α and IP10 the separate inhibitors had similar suppressive effects and both inhibitors were required for the best inhibition at 3 h post exposure. The inhibition was largely lost by 6 h for IP10 (Figure 7) and by 24 h for MIP1α (Table S2). IL6 inhibition was not significant at 3 h, likely due to the low response, but at 6 h suppression occurred and was dependent on the JNK inhibitor (Figure 7). At 24 h pi only the combined inhibitors continued to suppress production of TNFα, IL6, and MCP1 (Figure 8B, Table S2). IP10 and MIP1α production levels were equivalent to untreated cells by 24 h pi (Table S2). At 24 h the effect of the inhibitors on KC and G-CSF were slightly reversed as production was elevated by ERK, JNK or combined JNK/ERK inhibitor treatment but only the effect of ERK inhibition on KC reached significance (Figure 8B). IL1α was not detectable until 24 h post infection at which point it was strongly inhibited by ERK inhibitor, but not by JNK alone, and the combined inhibitors suppressed production further (Figure 8A). IL10 production was also not observed until 24 h pi and the concentrations produced were low compared to most other CK/CHKs at that time. The inhibitors effects were very different from the commonly observed pattern as IL10 production was increased by the ERK inhibitor and not altered by JNK or the combination.

**Figure 9:**
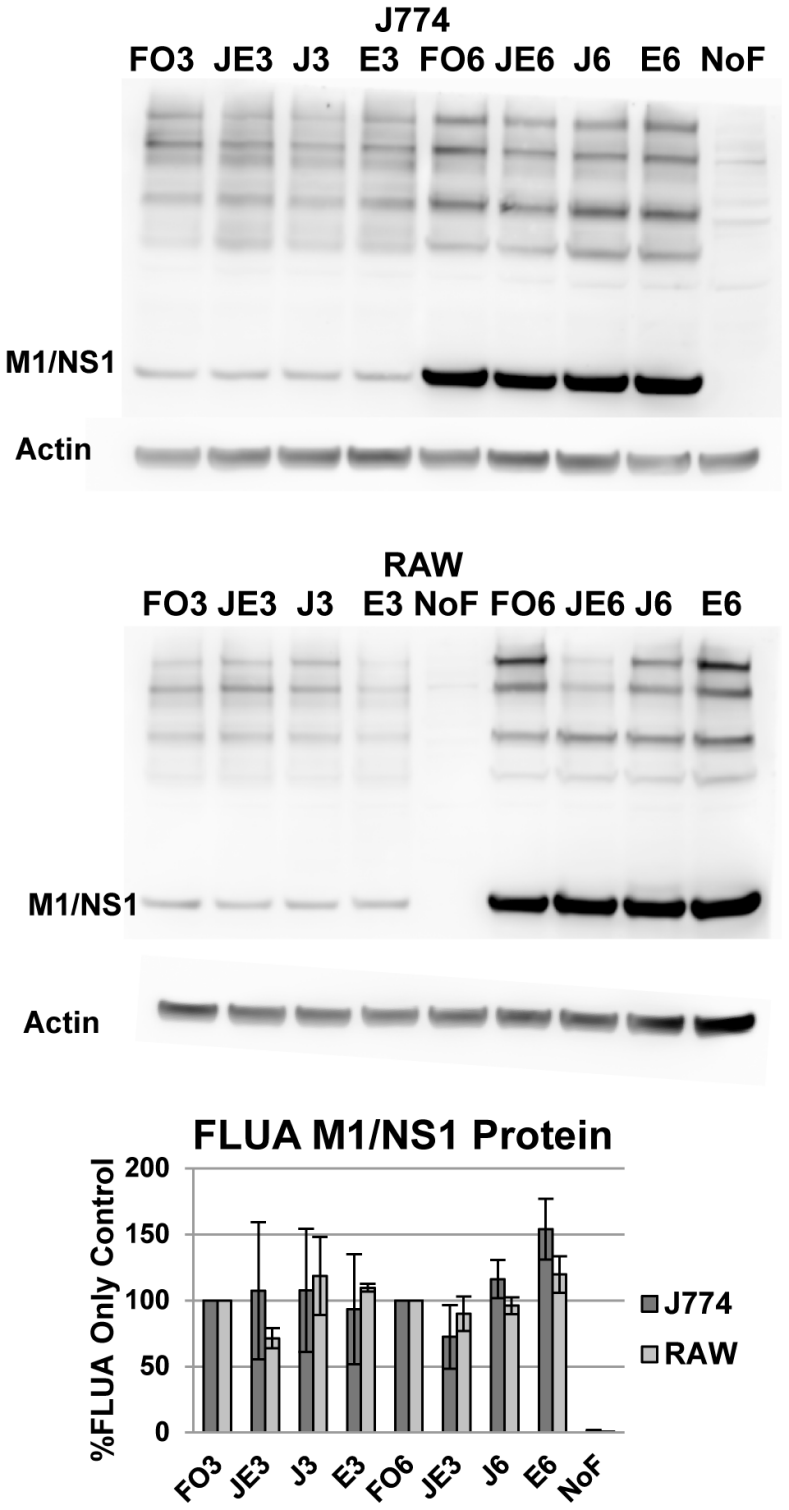
MAPK inhibitors alter CK/CHK production without reducing viral protein production in either cell type. As MAPK inhibition did not substantially block FLUA protein production the decreased CK/CHK production is not due to a direct anti-viral effect of the inhibitors. Representative Western blots of FLUA proteins. Densitometry values shown are the percent protein produced by the inhibited cells compared to the infected, uninhibited cells at the corresponding time points; mean percentage ± SEM, n = 3 independent experiments. FO3–FLUA only 3 h pi; JEI3–both JNK and ERK inhibition 3 h pi; JI3–JNK inhibition 3 h pi; EI3–ERK inhibition 3 h pi; NoF–mock infected cells with no inhibitors (treated with DMSO); FO6–FLUA only 6 h pi; JEI6–both JNK and ERK inhibition 6 h pi; JI6–JNK inhibition 6 h pi; EI6–ERK inhibition 6 h pi.

X-31 inactivated by UV, e.g. non-replicating FLUA, induced minimal TNFα in J774.A1 cells compared to live virus [43] and MAPKs have been shown to interfere with FLUA replication in non-monocytic cells [75]. We assayed X-31 protein production in JNK and ERK inhibited cells to ensure changes in cytokine production were not due to decreased viral entry or protein expression (Figure 9). By 3 h pi the standard profile of viral proteins were observed and by 6 h the expected increase in those bands was apparent both in cells that did not receive inhibitors and in those that did. There was no substantial decrease in production of viral proteins in the cells that received inhibitor compared to those that did not as judged by densitometry of virus specific bands.

## Discussion

In these experiments we examined the degree of activation and kinetics of cell signaling for both of the major NFκB pathways and the MAPK pathways thought to be important for proinflammatory CK/CHK production in response to FLUA. We also examined the levels and kinetics of a range of proinflammatory CK/CHKs produced, and the effects on that production caused by JNK and ERK inhibitors.

Our results show that these two monocyte cell lines demonstrate a very early and strong activation of JNK1/2 and ERK1/2 with large increases in phosphorylation/activation occurring as early as 15 minutes post FLUA exposure. Inhibitors of JNK and ERK, used singly and in combination, caused wide spread inhibition of CK/CHK production emphasizing the importance of MAPKs to these early responses. In contrast, markers of NFκB activation showed limited to no activation compared to controls at early time points. Based on the kinetics and magnitude of the MAPK responses and the effect their inhibitors have on CK/CHK production, the data are consistent with JNK and ERK having a major role in early CK/CHK production by macrophages.

We looked carefully at NFκB activation because it has long been thought to be crucial to pro-inflammatory CK/CHK production. FLUA is known to target pathways that lead to its activation, at least early in infection [68], [76]–[78]. Results shown here are consistent with those from PR8 infection of RAW 264.7 cells which showed no IκBα decrease up to 16 h pi [44], and from pH1N1 infection in swine cells for which only very late 16–32 h activation occurred [40] (X-31 is a re-assortment virus containing the H3N2 proteins of Aichi/68 but the internal proteins of PR8 [45]). In contrast an early 0.5 h pi activation of NFκB by H5N1 and H1N1 has been seen in human PBMC [38] but was not directly linked to early CK/CHK production. Using plasmid constructs, viral protein alone was shown to activate both the classical and the alternative NFκB pathway at later time points [23], [36], We saw neither effect here at our earlier time points perhaps because these macrophages have a non-productive infection [45], [51] and are starting to undergo apoptosis by the time viral proteins might be approaching sufficient levels [79], [80].

This study extends previous reports by demonstrating within a single study concurrent production of multiple CK/CHKs and provides additional details of the CK/CHK production kinetics. *In vivo*, cytokines and chemokines act in concert so the overall grouping of molecules in the response to a pathogen over time is important. We show that the macrophage cell lines J774.A1 and RAW 264.7 exposed to X-31 both produced TNFα, IL1α, IL6, IL10, IL13, MCP1, MIP1α, RANTES, KC, and IP10. RAW 264.7 cells also produced G-CSF. FLUA did not induce IL12, IL15, IL17, IL2, IL4, or IL5. The CK/CHK response was in agreement with existing murine (*in* *vitro* and *in vivo*) and human literature across multiple studies. Of the CK/CHK observed activated in this study, 10 out of 14 were identified in at least one murine *in vivo* study, 6 out of 9 in murine macrophages *in vitro*, and 9 out of 12 in human PBMCs. These points are illustrated in detail by the listing of CK/CHK and associated citations (Figure S1). Many of the previously published studies that have assayed more than 3 CK/CHKs at once have done so by measuring mRNA. While that information is certainly valuable, CK/CHKs are subject to substantial post-translational regulation so it is important to measure actual protein.

The MAPK inhibitor data show a clear link between the rapid activation of JNK and ERK and macrophage production of the 10 CK/CHKs activated by FLUA. The inhibitors generally reduced production but the specifics of the kinetics and the differences between the cells were complex. Generally in these cells JNK inhibition reduced the expression of most CK/CHKs as early as 3 h pi, with ERK inhibitors having a similar but lesser effect, and with a further reduction in the presence of the combined inhibitors. In some cases where neither single inhibitor had a strong effect, the combination did. At later time points for some CK/CHK, as early as 6 h, and for most (but not all) by 24 pi, the inhibitors’ effect on CK/CHK production was lost. It is not clear whether this was due to the inhibitors’ loss of efficacy over time or to the delayed activation of other pathways, i.e. p38 (Fig. 4C) or NFκB as seen by IκBβ degradation (Fig. 1B).

Understanding the severity of disease and pathogenesis produced by influenza infection depends on the type of cell exposed and the strain of virus involved. Although different influenza strains induce CK/CHK production at slower or greater rates and to higher or lower levels, the types of CK/CHK induced are very similar [10], [81]. Macrophages are particularly difficult to characterize because of the many different types of macrophages each with variant responses to pathogens. Alveolar macrophages have particular relevance to influenza infections because of their location in the lung. There is increasing evidence that alveolar macrophage responses show CK/CHK profiles more characteristic of alternately activated macrophages [81]–[85] and may play an important role in controlling the inflammatory response in the sensitive tissue of the lung [19]. Classically activated or infiltrating macrophages are located throughout the upper respiratory tract where respiratory viruses are often first encountered and arrive in great numbers after a pathogenic challenge. Thus they are also crucial to influenza clearance and pathology. Although originally not characterized as such RAW 264.7 and J774.A1 cells show what is now termed classical, versus alternate, activation for macrophages [71]. Both J774.A1 and RAW 264.7 cells were derived from BALB/C mice, but they were developed using different methodologies and are reported to have different levels of differentiation [52], [86], [87]. Differences between their responses were consistent with their difference in differentiation state. In general RAW 264.7 cells produced greater amounts of CK/CHKs sooner than J774.A1 cells.

These studies support previous suggestions that the use of MAPKs may have therapeutic value in influenza infections [24]. The avian H5N1 virus has been shown to target NFκB activation [62]. Understanding how alternative innate immune signaling proceeds in the face of that type of block without CK/CHK reduction has potential clinical relevance, particularly towards prophylactic treatment. Consistent with this idea, inhibition of ERK activation in the lungs of mice prior to infection with H7N7 or H5N2 isolates increased survival and lowered viral titers at 24 h [88]. The timing of such treatment is clearly important to balance the effects on viral titer, CK/CHK production and tissue damage. Mechanisms for controlling damage caused by inflammatory macrophages may be as crucial to promoting surviving as mechanisms for enhancing innate immune responses. Tight regulation of CK/CHK production is necessary to prevent tissue damage from an overactive response [10]. Many investigations into post FLUA signaling have emphasized later time points since treatment development is logically based on things that have an effect after symptoms appear. Early events that may have lasting effects on influenza induced pathogenesis also deserve consideration. Inhibiting the JNK and ERK pathways in macrophages prior to or very early after infection may lead to better control of FLUA pathogenesis.

Differences in viral isolates and titers, and in host genetic predisposition and prior experience all contribute to the degree of infection and any related pathology [89]–[91]. The study of one strain of virus as it interacts with one specific type of cell cannot hope to recapitulate the full innate immune response even during a strictly limited time frame. This simplified system does allow us to examine molecular events that might be obscured in the variant responses of individual hosts or even different cell type responses. It is of course a model *in vitro* system which will need to be tested in primary cells, *in vivo* and in humans. Nonetheless our particular combination of cell types and virus isolate allowed us to explore early CK/CHKs induction with limited NFκB participation. Using early time points to limit the components of the response under study and using a monoculture to eliminate feedback from other cell types, we show that even when the virus manages a substantial block on NFκB activation, the block does not prevent macrophages from mobilizing an immune response through the release of pro-inflammatory mediators using the activation of MAPK signaling pathways. At least in this one small battleground between the virus and the host, the host appears to be winning.

## Supporting Information

Figure S1 **Comparison of CK/CHKs Observed in this Study with Previous Reports.** For each CK/CHK the response in this study and responses reported *in vivo* for mice, for murine monocyte/macrophages *in vitro*, and for human peripheral blood monocytic cells exposed to various strains of FLUA are shown. Rose fill indicates that observations in the present study concur with literature reports. ND indicates not determined and grey fill Indicates no response observed from the J774.A1 or RAW 264.7 cells. Numbers correspond to the citation listing in this supplemental material.
(TIF)

Table S1 **Summary of CK/CHK Concentration Changes in Response to MAPK Inhibitors in J774.A1 Cells.** Data are the mean concentrations in pg/ml ± SEM at each time-point (3, 6, or 24 h, pi) for the cells treated with JNK and ERK inhibitors in combination (Both) and with each inhibitor individually compared to the amount produced by the uninhibited control cells in 3 independent experiments. Also shown are the levels of significance between the untreated and each of the treated samples using multiple two tailed Student’s t-tests. The tests were two tailed as *a priori* it was not assumed that the MAPK inhibitors would be inhibitory or stimulatory for all CK/CHK at all times pi. Significance levels are indicated as: P<0.001 (****); P<0.01 (***); P<0.05 (**); P<0.2 (*). P<0.2 is shown to mark those changes where the absolute values showed strong likely inhibitor effects by inspection but the P<0.05 level of significance was not reached.
(TIF)

Table S2 **Summary of CK/CHK Concentration Changes in Response to MAPK Inhibitors in RAW 264.7 Cells.** Data are the mean concentrations in pg/ml ± SEM at each time-point (3, 6, or 24 h, pi) for the cells treated with JNK and ERK inhibitors in combination (Both) and with each inhibitor individually compared to the amount produced by the uninhibited control cells in 3 independent experiments. Also shown are the levels of significance between the untreated and each of the treated samples using multiple two tailed Student’s t-tests. The tests were two tailed as *a priori* it was not assumed that the MAPK inhibitors would be inhibitory or stimulatory for all CK/CHK at all times pi. Significance levels are indicated as: P<0.001 (****); P<0.01 (***); P<0.05 (**); P<0.2 (*). P<0.2 is shown to mark those changes where the absolute values showed strong likely inhibitor effects by inspection but the P<0.05 level of significance was not reached.
(TIF)
