# Supplementary figures and images for: Early Activation of MAP Kinases by Influenza A Virus X-31 in Murine Macrophage Cell Lines
Source: PLoS One. 2014 Aug 28;9(8):e105385. doi: 10.1371/journal.pone.0105385 (PMC4148262; doi:10.1371/journal.pone.0105385)

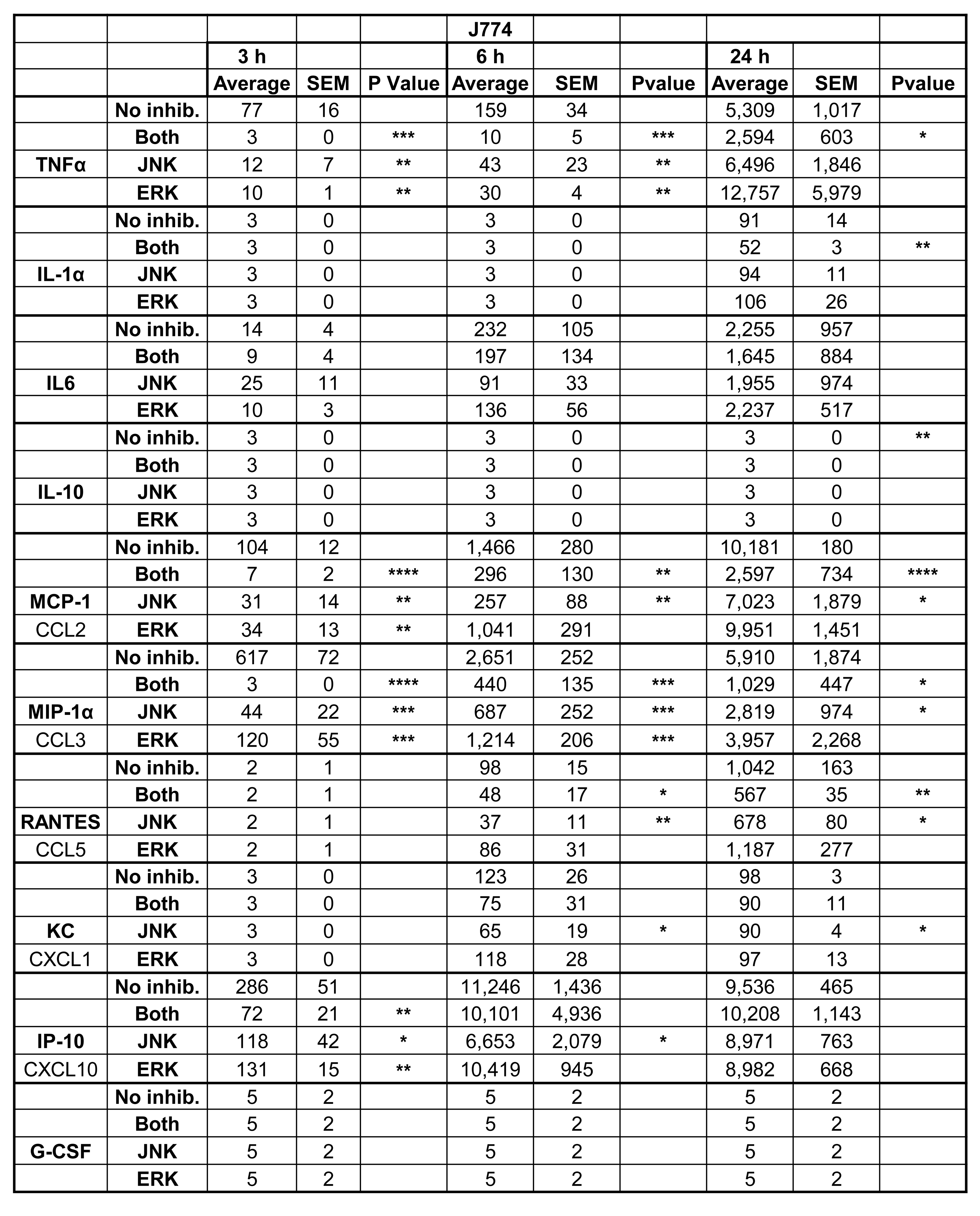

Supplement: Table S1 — Summary of CK/CHK Concentration Changes in Response to MAPK Inhibitors in J774.A1 Cells. Data are the mean concentrations in pg/ml ± SEM at each time-point (3, 6, or 24 h, pi) for the cells treated with JNK and ERK inhibitors in combination (Both) and with each inhibitor individually compared to the amount produced by the uninhibited control cells in 3 independent experiments. Also shown are the levels of significance between the untreated and each of the treated samples using multiple two tailed Student’s t-tests. The tests were two tailed as a priori it was not assumed that the MAPK inhibitors would be inhibitory or stimulatory for all CK/CHK at all times pi. Significance levels are indicated as: P<0.001 (****); P<0.01 (***); P<0.05 (**); P<0.2 (*). P<0.2 is shown to mark those changes where the absolute values showed strong likely inhibitor effects by inspection but the P<0.05 level of significance was not reached. (TIF) [file pone.0105385.s002.tif]

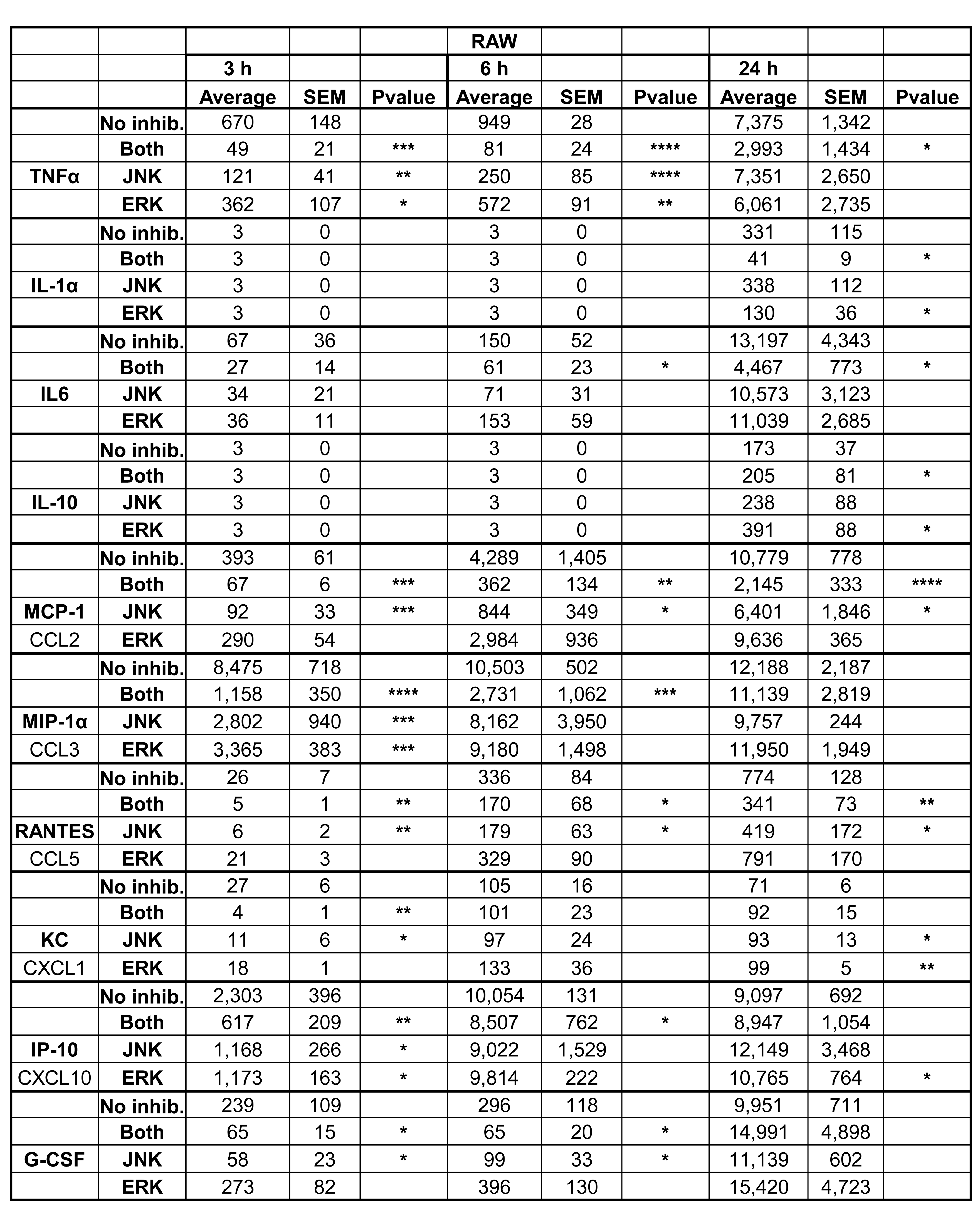

Supplement: Table S2 — Summary of CK/CHK Concentration Changes in Response to MAPK Inhibitors in RAW 264.7 Cells. Data are the mean concentrations in pg/ml ± SEM at each time-point (3, 6, or 24 h, pi) for the cells treated with JNK and ERK inhibitors in combination (Both) and with each inhibitor individually compared to the amount produced by the uninhibited control cells in 3 independent experiments. Also shown are the levels of significance between the untreated and each of the treated samples using multiple two tailed Student’s t-tests. The tests were two tailed as a priori it was not assumed that the MAPK inhibitors would be inhibitory or stimulatory for all CK/CHK at all times pi. Significance levels are indicated as: P<0.001 (****); P<0.01 (***); P<0.05 (**); P<0.2 (*). P<0.2 is shown to mark those changes where the absolute values showed strong likely inhibitor effects by inspection but the P<0.05 level of significance was not reached. (TIF) [file pone.0105385.s003.tif]
